# Up-Regulation of Kin17 Is Essential for Proliferation of Breast Cancer

**DOI:** 10.1371/journal.pone.0025343

**Published:** 2011-09-29

**Authors:** Tao Zeng, Hongyi Gao, Pei Yu, Heng He, Xiaoming Ouyang, Lijuan Deng, Yan Zhang

**Affiliations:** 1 Key Laboratory of Gene Engineering of the Ministry of Education, State Key Laboratory of Biocontrol, School of Life Sciences, Sun Yat-sen University, Guangzhou, People's Republic of China; 2 Department of Pathology, Guangdong Women and Children's Hospital and Health Institute, Guangzhou, People's Republic of China; 3 Department of Pathology, The Second Affiliated Hospital of Guangzhou Medical University, Guangzhou, People's Republic of China; Universita' di Milano, Italy

## Abstract

**Background:**

Kin17 is ubiquitously expressed at low levels in human tissue and participates in DNA replication, DNA repair and cell cycle control. Breast cancer cells are characterized by enabling replicative immortality and accumulated DNA damage. However, whether kin17 contributes to breast carcinogenesis remains unknown.

**Methodology/Principal Findings:**

In this study, we show for the first time that kin17 is an important molecule related to breast cancer. Our results show that kin17 expression was markedly increased in clinical breast tumors and was associated with tumor grade, Ki-67 expression, p53 mutation status and progesterone receptor expression, which were assessed in a clinicopathologic characteristics review. Knockdown of kin17 inhibited DNA replication and repair, blocked cell cycle progression and inhibited anchorage-independent growth, while increasing sensitivity to chemotherapy in breast cancer cells. Moreover, kin17 silencing decreased EGF-stimulated cell growth. Furthermore, overexpression of kin17 promoted DNA replication and cell proliferation in MCF-10A.

**Conclusions/Significance:**

Our findings indicate that up-regulation of kin17 is strongly associated with cellular proliferation, DNA replication, DNA damage response and breast cancer development. The increased level of kin17 was not only a consequence of immortalization but also associated with tumorigenesis. Therefore, kin17 could be a novel therapeutic target for inhibiting cell growth in breast cancer.

## Introduction

Breast cancer is the most commonly diagnosed cancer and the second leading cause of cancer-related death among women [1]. Although progress has been made in reducing the incidence and mortality of breast cancer, disseminated metastasis still remains incurable [2]. Furthermore, a number of early-stage breast cancer patients relapse, regardless of the adjuvant treatment given. Substantial efforts have been made to understand the molecular bases of the disease to support new discoveries in early detection and effective treatment.

Kin17 is a gene that is highly conserved across evolution. The structures of kin17 and *E. coli* RecA proteins share an antigenic determinant that is located in the core of kin17 protein and in the C-terminal end of RecA protein [3]. Kin17 is ubiquitously expressed in mammals and is associated with very important physiological functions. In humans, it is generally expressed at extremely low levels in all tissues and organs, except for in heart, skeletal muscle and testis [3]. Kin17 binds preferentially to the curved DNA found at hot-spots of illegitimate recombination in eukaryotic chromosomes and has a tandem SH3 domain that participates in RNA binding [4], [5]. Kin17 was determined to be a component of a multiprotein DNA replication complex and to be associated with mammalian replication origins, mRNA processing, transcription and cell cycle regulation [6], [7]. It also participates in the general response to genotoxic stress and is increased following DNA damage produced by UVC or ionizing radiation, depending on the integrity of the human global genome repair machinery [3], [8], [9]. Kin17 acts as a DNA maintenance protein and helps to overcome perturbations in DNA replication produced by unrepaired lesions [10]. Breast cancer cells are characterized by uncontrolled growth, unlimited replication potential and accumulated DNA damage [11]–[13]; therefore, investigating the function of kin17 in breast cancers could elucidate some of the mechanisms driving breast cancer growth as well as the implications of these mechanisms for treatment strategies.

In this study, we examined kin17 expression in benign and malignant breast tumors. The strong kin17 expression that we observed in neoplastic cells and advanced tumors suggested a potential role for kin17 in breast tumorigenesis. Knockdown of kin17 inhibited DNA replication and repair, reduced tumor cell growth and colony formation. Our work is the first to demonstrate that kin17 plays a significant role in breast cancer pathogenesis and progression.

## Results

### Kin17 expression in breast tumors and cells

**Figure 1:**
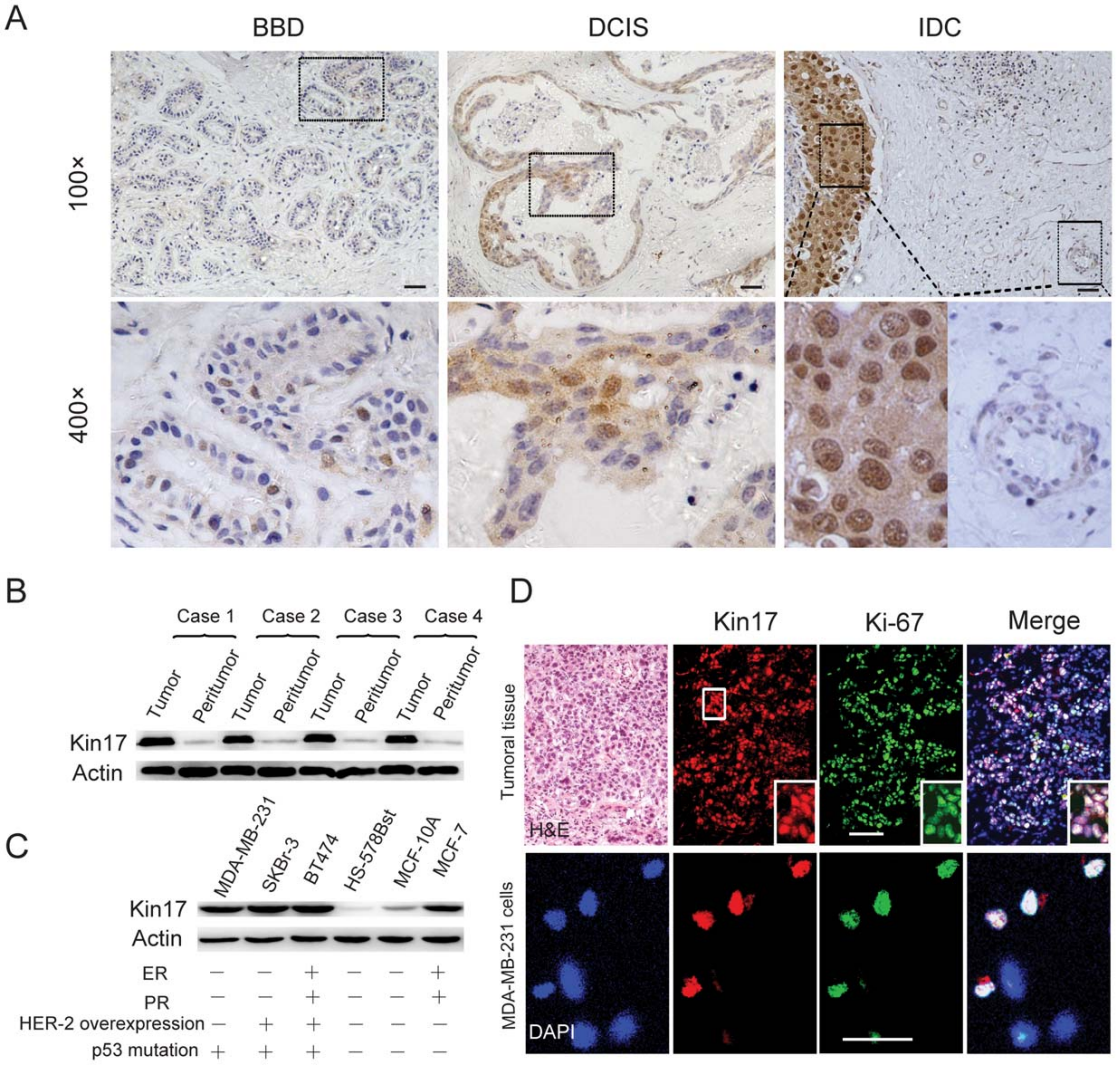
Kin17 expression in clinical breast tumors and cell lines. (A) Kin17 expression in BBD, DCIS and IDC tissues. The negative controls for each tissue did not show staining (Figure S1). Scale bars = 30 µm. (B) Western blot of kin17 in IDC tissues and peritumoral tissues. (C) Western blots of kin17 in breast cancer cells and normal cells. The expression status of ER, PR and HER-2, in addition to the p53 mutation status, is listed for each cell type [37]. (D) Dual staining of kin17 and Ki-67 in clinical IDC samples and MDA-MB-231 cells. Scale bars = 30 µm.

To explore the function of kin17 in breast cancers, we first investigated kin17 expression in breast tumor tissues and peritumoral tissues. We examined 127 samples from patients with breast disease, including 40 benign breast diseases (BBD) samples, 22 ductal carcinoma in situ (DCIS) samples and 65 invasive ductal carcinoma (IDC) samples, by immunohistochemistry. Ninety percent (36 out of 40) of the BBD showed weak staining, while 81.8% (18 out of 22) of DCIS expressed kin17 at low levels; 18.2% (4 out of 22) had higher levels. Notably, 43.1% (28 out of 65) of the IDC samples displayed very strong nuclear staining and weak cytoplasmic staining (Table 1, Figure 1A and Figure S1). Kin17 expression was significantly higher in IDC than in BBD (*p* = 0.0001) or DCIS tissues (*p* = 0.036). In DCIS tissues, the kin17 expression was slightly higher than in benign lesions; however, there was no statistical differences between the two (*p* = 0.601). In contrast, the peritumoral epithelium displayed very weak or negative staining (Figure 1A). Thus, the kin17 immunostaining in IDC was stronger than that in their peritumoral counterparts (*p* = 0.0001, Table 1). It seems that kin17 expression is markedly up-regulated in breast cancers. To further confirm our hypothesis, we compared kin17 expression in IDC tissues and their peritumoral counterparts by western blot. We found that the kin17 expression level was significantly higher in tumors than in their peritumoral counterparts (Figure 1B).

**Table 1:** Association between kin17 staining and groups of breast disease.

		Kin17 expression		
Groups	n (%)	Low	High	*P* value
BBD	40(100)	36(90.0)	4(10.0)	0.0002^a^ *
DCIS	22(100)	18(81.8)	4(18.2)	
IDC	65(100)	37(56.9)	28(43.1)	
BBD vs. IDC				0.0001^b^ *
BBD vs. DCIS				0.601^c^
DCIS vs. IDC				0.036^b^ *
Peritumoral tissues in IDC	52(100)	51 (98.1)	1 (1.9)	0.0001^b^ *
Intratumoral tissues in IDC	65(100)	37 (56.9)	28 (43.1)	

Abbreviation: BBD, benign breast diseases; DCIS, Ductal carcinoma in situ; IDC, Invasive ductal carcinoma.

a Cochran-Mantel-Haenszel test,

b Pearson Chi-Square test,

c Continuity correction test,

*statistically significant.

We also examined kin17 expression in breast epithelial cells. We collected six types of breast cells, including four breast cancer cell lines (MDA-MB-231, SKBr-3, BT474 and MCF-7), the immortalized non-tumorigenic cell line MCF-10A and the normal breast epithelial cell Hs578Bst. Kin17 expression was extremely low in Hs578Bst cells, while all four breast cancer cell lines displayed high kin17 expression. The MCF-10A cells had a slightly higher level of kin17 than the Hs578Bst cells, but this level was still markedly lower than that in the four cancer cells (Figure 1C). In addition, we found that kin17 was weakly expressed in vascular endothelial cells in benign tumors and DCIS tissues but strongly expressed in the vascular endothelial cells within IDC tissues (*p* = 0.011, Figure S2). It seems that kin17 expression was elevated in the vascular endothelial cells located in malignant tumor tissues.

### Association of kin17 expression with clinicopathologic parameters

We reviewed the clinical characteristics of 65 patients with IDC and examined the correlation of baseline characteristics with the kin17 expression level. Kin17 expression level was significantly associated with tumor grade (*p* = 0.049, Table 2), luminal B subtype (ER^+^PR^+^HER2^+^, *p* = 0.016) and Ki-67 expression (*p* = 0.046), but there were no significant association with age, tumor size, tumor stage, estrogen receptor (ER) status, HER2 status or VEGF expression by univariate analysis (Table 2). To further confirm the association between kin17 and Ki-67, frozen IDC tissues were used for dual-labeled immunofluorescence analysis with kin17 and Ki-67 antibodies. Similar to Ki-67, kin17 was mostly nuclear located and strongly stained. Almost all of the Ki-67-positive cells were kin17-positive (Figure 1D). The same result was obtained in MDA-MB-231 cells, where kin17 and Ki-67 were obviously co-expressed in the nucleus (Figure 1D). Ki-67 is regarded as a marker for cell proliferation [14], so co-expression of kin17 and Ki-67 in tumor tissues and cells suggests that kin17 may also be related to cellular proliferation.

**Table 2:** Association between clinicopathologic variables and kin17 expression in the 65 patients with IDC.

		Kin17 expression		
Variales	n(%)	Low	High	*P* value
Age — yr	Mean 43.5	43.5±7.7	43.5±8.5	0.8945^a^
Tumour size —n(%)	Mean 30.6 mm			
≤30.6 mm	42 (64.6)	24 (64.9)	18 (64.3)	0.961^b^
>30.6 mm	23 (35.4)	13 (35.1)	10 (35.7)	
lymph nodes metastasis				
No	41(63.1)	25(67.6)	16(57.1)	0.388^b^
Yes	24(36.9)	12(32.4)	12(42.9)	
Tumour grade—n(%)				
1	14 (21.5)	12 (32.4)	2 (7.1)	0.049^b^ *
2	35 (53.9)	17 (46.0)	18 (64.3)	
3	16(24.6)	8(21.6)	8(28.6)	
Tumor stage—n(%)				
I	15 (23.1)	10 (27.0)	5 (17.9)	0.658^b^
II	40 (61.5)	22 (59.5)	18 (64.2)	
III	10(15.4)	5(13.5)	5(17.9)	
HER2 status—n(%)				
Negative	25 (38.5)	15 (40.5)	10 (35.7)	0.692^b^
Positive	40 (61.5)	22 (59.5)	18 (64.3)	
Luminal A(ER+PR+HER2−)—n(%)				
No	52(80.0)	30(81.1)	22(78.6)	0.802^b^
Yes	13(20.0	7(18.9)	6(21.4)	
Luminal B(ER+PR+HER2+)—n(%)				
No	51(78.5)	33(89.2)	18(64.3)	0.016^b^ *
Yes	14(21.5)	4(10.8)	10(35.7)	
HER2 overexpression subtype (ER−PR−HER2+)—n(%)				
No	50 (76.9)	30 (81.1)	20 (71.4)	0.360^b^
Yes	15 (23.1)	7 (18.9)	8 (28.6)	
Basal-like subtype (ER−PR−HER2−)—n(%)				
No	54(78.5)	29(78.4)	25(89.3)	0.408^c^
Yes	11(21.5)	8(21.6)	3(10.7)	
p53 status—n(%)				
Wild	43 (66.2)	29 (78.4)	14 (50)	0.017^b^ *
Mutant	22 (33.8)	8 (21.6)	14 (50)	
VEGF expression—n(%)				
Low	32 (56.1)	16 (51.6)	16 (61.5)	0.452^b^
High	25 (43.9)	15 (48.4)	10 (38.5)	
Ki-67 status—n(%)				
Negative	17 (34.7)	12 (48.0)	5 (20.8)	0.046^b^ *
Positive	32 (65.3)	13 (52.0)	19 (79.2)	

a Mann-whitney u test,

b Pearson Chi-Square test,

c Continuity correction test,

*statistically significant.

In addition, we also found a significant association between kin17 expression and progesterone receptor (PR) expression (*p* = 0.046) as well as p53 mutation status (*p* = 0.017). The relationship among these factors needs to be further explored.

### Kin17 is associated with cell proliferation, DNA replication, cell cycle progression and the regulation of cell morphology

**Figure 2:**
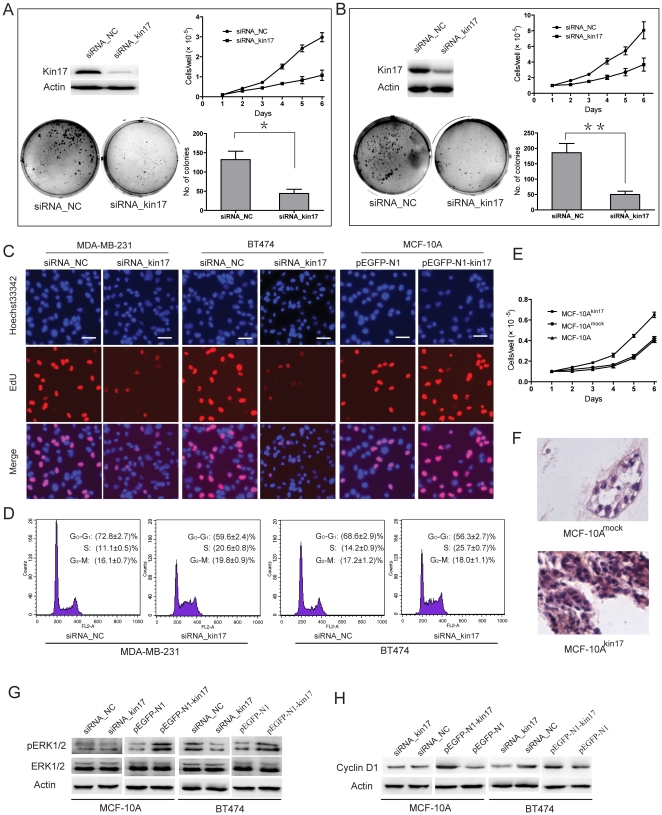
Association of kin17 expression with cell proliferation, colony formation, DNA replication and cell cycle progression. MDA-MB-231 (A) and BT474 cells (B) transfected with siRNA_kin17 showed reduced growth rate and colony formation compared to the controls. * *p*<0.05, ** *p*<0.01. (C) Knockdown of kin17 expression inhibited DNA replication in MDA-MB-231 and BT474 cells compared to controls as determined by the EdU incorporation assay. Elevated expression of kin17 increased DNA replication in MCF-10A cells. (D) Cell cycle phase distributions were analyzed in a FACScalibur flowcytometer. These experiments were repeated 3 times, and the symbols represent the mean values of triplicate tests (mean ± SD). (E) Overexpression of kin17 following transfection with pEGFP-N1-kin17 promoted cell proliferation in MCF-10A cells. Cells positive for the EGFP-N1-kin17 were designated MCF-10A^kin17^, while cells transfected with pEGFP-N1 vector were designated MCF-10A^mock^. (F) Overexpression of kin17 disrupted acinar organization and blocked luminal clearance in MCF-10A cells. Western blotting analysis of autophosphorylation of ERK1/2 (G) and cyclin D1 expression (H). The experiment was independently repeated at least 3 times.

In order to analyze the effects of kin17 on proliferative and morphogenic phenotypes of breast epithelial cells and breast cancer cells, we knocked down endogenous kin17 in MDA-MB-231 (Figure 2A) and BT474 cells (Figure 2B) with siRNA_kin17. Kin17 knockdown significantly inhibited the proliferation of MDA-MB-231 (*p*<0.01) and BT474 cells (*p*<0.05), however, had little effect on the proliferation of MCF-10A cells (Figure S3). After knockdown of kin17, the colony number in soft agar was decreased by 2.0 fold in MDA-MB-231 and 2.7 fold in BT474 cells (*p*<0.05 and *p*<0.01, respectively). To investigate the mechanisms underlying altered cell growth, EdU retention assays were performed to examine the regulatory effect of kin17 on DNA replication. Following transfection with siRNA_kin17, the percentage of EdU-positive cells was reduced in MDA-MB-231 and BT474 cells compared to the controls (Figure 2C). Moreover, the MDA-MB-231 and BT474 cells that were transfected with siRNA_kin17 had a smaller part of the population in G_0_-G_1_ phase and an enhanced proportion in S phase (Figure 2D). It seems that knockdown of kin17 resulted in cells that were blocked in S phase and could not enter into G_2_ phase for cell division.

pEGFP-N1-kin17 was transfected into MCF-10A cells to investigate the effect of kin17 on non-tumorigenic cells. Our results showed that the proliferative rate of MCF-10A^mock^ was similar to parental cells. While MCF-10A^kin17^ cells showed a higher proliferative capacity, as determined by the EdU retention assay, compared to MCF-10A^mock^cells in 2-dimensional (2-D) cultures (Figure 2C and Figure 2E). Thus up-regulation of kin17 promoted the proliferation of breast non-tumorigenic cells. MCF-10A^mock^ cells formed branching tubules and lumen in 3-D matrigel cultures. However, MCF-10A^kin17^ cells proliferated quickly and rarely formed branching tube-like and luminal structures. The small structures formed by MCF-10A^kin17^ cells never produced outgrowths that reached the size generated by MCF-10A^mock^ cells (Figure 2F). Additionally, up-regulation of kin17 increased ERK1/2 autophosphorylation and cylin D1 expression in MCF-10A and BT474 cells. While kin17 knockdown decreased ERK1/2 autophosphorylation and cyclin D1 expression in BT474 cells. However, ERK1/2 autophosphorylation and cyclin D1 expression did not change when kin17 expression was down-regulated in MCF-10A cells (Figure 2G and Figure 2H).

### Elevated kin17 expression was required for DNA repair in breast cancer cells

**Figure 3:**
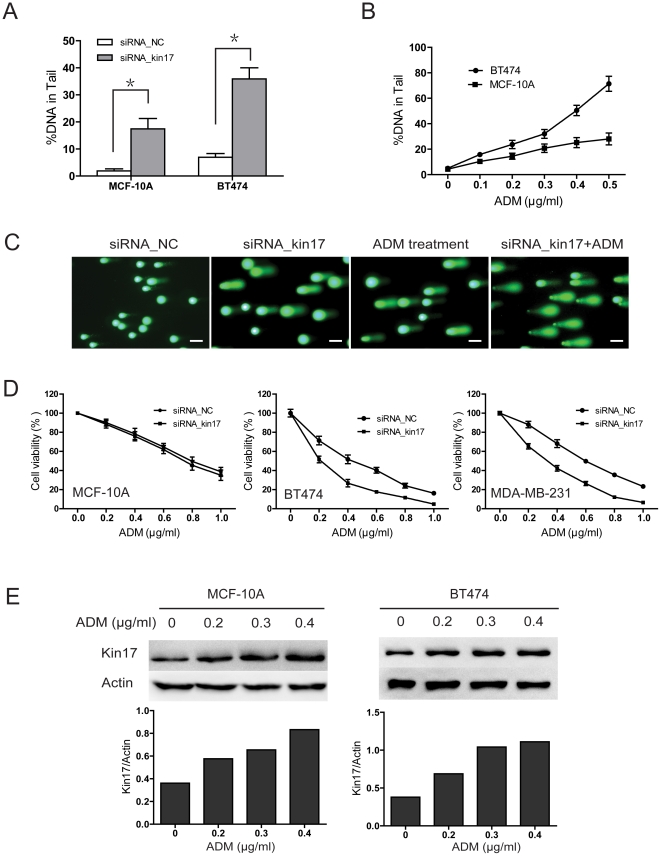
Kin17 is associated with DNA damage in breast cells. (A) Knockdown of kin17 increased DNA damage in MCF-10A and BT474 cells. (B) The cells were treated with ADM at the indicated concentrations for 24 hr, and analyzed using the comet assay kit. (C) Representative images of DNA damage in BT474 cells. The cells were transfected with siRNA_kin17 or treated with ADM (0.3 µg/ml) for 24 hr. Scale bars = 50 µm. (D) The effect of kin17 knockdown on the chemosensitivity to ADM in MCF-10A, BT474 and MDA-MB-231 cells (*p*<0.05). (E) The cells were treated with ADM at the indicated concentrations for 48 hr, and then kin17 expression was analyzed by western blot.

DNA damage is elevated in cancer cells due to carcinogenic exposure, telomere shortening and hypoxia [15]. An increased number of DNA lesions results in cell cycle arrest and cell death due to the activation of DNA damage repair pathways [11]. We hypothesized that elevation of kin17 would be necessary for DNA repair in breast cancer cells. In this study, knockdown of kin17 resulted in DNA damage in both MCF-10A and BT474 cells. However, more DNA damage was detected in the BT474 than in the MCF-10A cells after kin17 was knocked down (Figure 3A). We also treated cells with adriamycin (ADM), an anticancer drug that is commonly used in breast cancer, to induce DNA damage. The main forms of DNA damage induced by ADM are DNA double-strand breaks, DNA single strand breaks, alkali labile sites, and ADM–DNA adducts [16], [17]. Alkaline comet assay has been testified as an effective method to detect the DNA damage induced by ADM [18], [19]. Compared to the cells without ADM treatment, a strong induction of DNA damage was detected in the ADM-treated cancer cells, and more DNA damage appeared in the BT474 than in the MCF-10A cells (Figure 3B). Furthermore, siRNA_kin17-transfected breast cancer cells were much more sensitive to ADM treatment (Figure 3C and Figure 3D). However, kin17 silencing had no abvious effect on the chemosensitivity to ADM in MCF-10A cells (Figure 3D). In parallel with DNA damage, kin17 expression in MCF-10A and BT474 increased after ADM treatment (Figure 3E).

### Kin17 is required for EGF-stimulated cell growth

**Figure 4:**
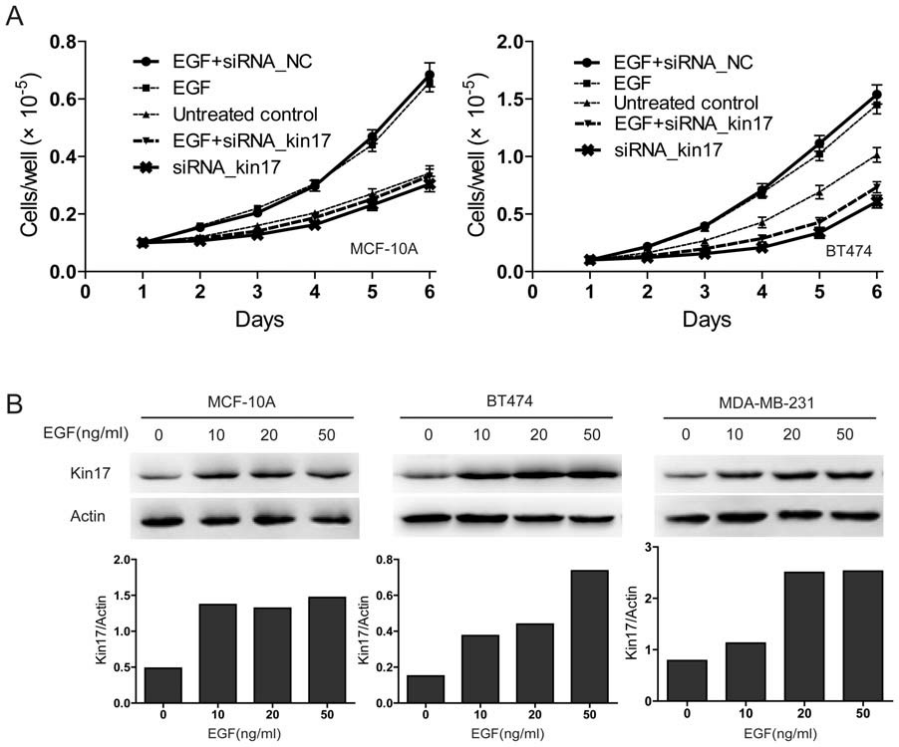
Kin17 is associated with an EGF response. (A) Kin17 silencing inhibited EGF-stimulated cell growth in MCF-10A and BT474 cells. *p*<0.05. (B) The cells were treated with recombinant human EGF for 48 hr, and kin17 expression was detected by western blot.

Epidermal growth factor (EGF) is an important growth factor associated with cell proliferation and tumorigenesis in breast cancer [20], [21]. To determine if kin17 plays a role in the increased cell proliferation caused by EGF, we knocked down kin17 and then stimulated cells with EGF. Compared to the cells transfected with siRNA_NC, EGF had little effect on the cells transfected with siRNA_kin17 (Figure 4A). In addition, stimulation with EGF resulted in an elevation in kin17 expression in MDA-MB-231, BT474 and MCF-10A cells (Figure 4B). These results suggest that kin17 is required for proliferation signaling initiated by EGF and that EGF can elevate kin17 expression.

## Discussion

Previous work has shown that human fibroblasts immortalized with SV40 have an elevated level of kin17 protein compared to normal diploid human fibroblasts [22]. In this study, we found that kin17 expression is dramatically increased in clinical breast cancer samples and breast cancer cells. In clinical samples, kin17 expression was significantly higher in IDC than in BBD and DCIS tissues. In breast cell lines, kin17 expression was higher in immortalized MCF-10A cells compared to normal Hs578Bst breast cells. Importantly, kin17 expression was higher in breast cancer cells compared to Hs578Bst and MCF10A cells. These results suggest that kin17 expression gradually increases as breast cancer advances from early- to late-stage disease. Additionally, increased kin17 expression was not only a consequence of the immortalized phenotype, but it was also associated with tumorigenesis.

Kannouche et al. reported that serum-stimulated mouse fibroblasts displayed a 5-fold higher expression of kin17 mRNA and increased cell growth compared to the cells without serum stimulation [23]. Our results agree with this study, and several lines of evidence suggest that up-regulation of kin17 is essential for mitogenesis. First, the level of kin17 expression was significantly associated with breast cancer grade and Ki-67 expression. High-grade breast cancer grows quickly and aggressively, and Ki67 serves as a marker of cell proliferation. Thus, elevated kin 17 expression is likely associated with breast cancer cell proliferation. Second, suppression of kin17 blocked cell cycle progression and inhibited growth in breast cancer cells. Finally, up-regulation of kin17 in breast epithelial cells promoted cell proliferation and influenced cell-cell interactions and differentiation. Collectively, these data suggest a role for kin17 in cellular proliferation.

Kin17 appears to play a central role in DNA replication and repair. Kin17 colocalizes with multiple replication proteins, including RPA, PCNA and DNA polymerase-α [7]. Specific monoclonal antibodies that are targeted against kin17 reduce cellular replication activity [7]. UVC irradiation induces the nucleoplasmic redistribution of kin17 protein, indicating a link between the intranuclear distribution of kin17 protein and the DNA damage response [3]. γ-irradiation increases the amount of kin17 protein bound to DNA and localizes kin17 protein to large nucleoplasmic foci [10]. Breast cancer cells exhibit replicative immortality [13] and a high level of endogenous replication lesions [12]. Our results showed that knockdown of kin17 in breast cancer cells inhibited DNA replication, aggravated DNA damage and blocked cells in the S phase of the cell cycle. Elevated kin17 expression appears to be required for DNA replication and repair and to prevent cell cycle arrest.

The signaling pathway through which kin17 promotes cell proliferation and tumorigenesis is unknown. Cyclin D1 is one of the key regulators of the G1/S transition [24], and gene amplification or overexpression of cyclin D1 is associated with cell growth and tumorigenesis in breast cancer [25]. Cyclin D1 expression is induced by various growth factors that are activated via the Ras/MEK/ERK pathway [26]. We found that kin17 increased cyclin D1 protein expression and constitutive phosphorylation of ERK1/2 in MCF-10A and BT474 cells. Suppression of kin17 blocked cell cycle progression and decreased the expression of cyclin D1 and ERK1/2 activity in breast cancer cells. These results suggest that ERK1/2 and cyclin D1 may be involved in the kin17 signaling cascade. p53 mutations have been identified in many types of cancer [27], and such mutations serve as the most powerful prognostic markers in breast cancer [28]. A significant association between increased kin17 expression and p53 mutations in our study would strongly suggest that kin17 is involved in breast cancer progression.

In recent years, a host of new rationally designed, molecularly targeted cancer therapies have been introduced as novel treatments, with the aims of improving the cure rate and reducing cytotoxicity in normal cells [29]. Breast cancer is a heterogeneous disease, with various subtypes exhibiting different susceptibilities to anticancer drugs. Therefore, therapies targeting specific breast cancer subtypes are recommended for effective treatment [30]. ER or PR positive patients show better responses to hormonal therapy than ER/PR negative patients [31], and the HER-2 monoclonal antibody trastuzumab has demonstrated efficacy in patients overexpressing HER-2 [32]. However, only 20%–30% of breast cancers are HER-2 positive, and some of the patients overexpressing HER-2 exhibit resistance to trastuzumab [33]. Triple-negative (ER^−^PR^−^HER2^−^) breast cancer patients, which accounts for 10–20% of all breast cancers, are usually of high histological grade and are accompanied by aggressive clinical symptoms, with shorter time to recurrences and death; these patients lack effective tailored therapies [34]. Interestingly, triple-negative patients frequently express EGFR [35]. EGFR signaling plays an important role in cell proliferation and tumorigenesis in a variety of cancers, including breast cancer [36]. Overexpression of EGFR protein occurs in 16–36% of breast cancers. Several therapies targeting EGFR (cetuximab, gefitinib and erlotinib) are in clinical use for advanced breast cancer. However, most of the clinical trials have not succeeded [36]. In the present study, MDA-MB-231 cells are triple-negative [33], and BT474 cells are ER^+^PR^+^HER2^+^ [37], down-regulation of kin17 inhibited cell growth and increased chemosensitivity to ADM in both MDA-MB-231 and BT474 cells, suggesting that therapy that targets kin17 is not limited by clinical immunophenotype. Moreover, silencing kin17 expression inhibited proliferation signaling initiated by EGF. Thus, we proposed that combination of kin17 knocking down with ADM treatment would reduce the dose of ADM, so as to lessen the drug resistance and toxicity of ADM in conventional treatment. In addition, kin17 knockdown displayed weak inhibition on proliferation of MCF-10A, and kin17 silencing showed little effect on ERK1/2 autophosphorylation, cyclin D1 expression and chemosensitivity to ADM in MCF-10A cells. Thus, kin17 silencing would be promising for breast cancer treatment. Furthermore, we show that kin17 is necessary for EGF-stimulated cell growth, therefore, treatment targeting kin17 could be combined with neutral EGFR antibody therapy to treat breast cancer.

In conclusion, in the present study, we make the novel proposal that kin17 could be a useful target for breast cancer therapy. This serves as a first step in characterizing kin17 at the molecular level using *in vitro* techniques. These data can be extended *in vivo* models to further elucidate the role of kin17 in cell proliferation and carcinogenesis.

## Materials and Methods

### Patients and specimens

Sixty-five IDC samples, 22 DCIS samples and 40 BBD samples were obtained and pathologically confirmed at the Guangdong Women and Children's Hospital and Health Institute. The 127 patients from whom the samples were derived underwent curative resection between 2003 and 2009. The clinical characteristics of all these patients are summarized in Table 2. Clinical stages were classified according to the International Union against Cancer. Fresh IDC and peritumoral tissues (at least 3 cm away from the tumor margin) from 4 patients were used for kin17 expression analysis. Frozen tumor tissues from 12 patients with IDC were used for the double-labeled immunofluorescence analysis. All samples were anonymously coded in accordance with local ethical guidelines, and written informed consent was obtained. This study was approved by the Review Board of Guangdong Women and Children's Hospital and Health Institute.

### Immunohistochemistry

Paraffin sections (4 µm thick) were deparaffinized and rehydrated followed by immersing in 3% H_2_O_2_ to quench endogenous peroxidase activity. Then, the sections were treated with 0.01 M citrate buffer (pH 6.0) and blocked with 5% normal goat serum followed by incubation with monoclonal anti-kin17 antibody (1∶100; Santa Cruz, k58), monoclonal anti-VEGF antibody (1∶100; Dako), monoclonal anti-ki-67 antibody (1∶100; Dako), monoclonal anti-estrogen receptors antibody (1∶100; Dako), monoclonal anti-progestrone receptors antibody (1∶100; Dako), polyclonal anti-HER2 rabbit antibody (1∶100; Dako), monoclonal anti-p53 antibody (1∶100; Dako) overnight at 4°C, respectively. These primary antibodies were diluted in PBS buffer containing 5% normal goat serum. The negative control for each slide was incubated with 5% normal goat serum without primary antibody. The sections were then incubated with HRP-conjugated anti-mouse or anti-rabbit IgG for 45 min at room temperature and revealed with a ChemMate™ Envision™ Dectection Kit (DAKO). The stained slides were scored by two investigators, who were unaware of the clinical diagnosis, according to the method described by Merritt W M et al [38]. A staining score (intensity of staining×percentage of cells stained) of >100 was defined as high expression and a staining score of ≤100 was defined as low expression. The specificity and selectivity of kin17 antibody (K58) have been determined by Miccoli L [22] and Biard DS [1].

### Cell culture

The human breast cancer cell lines MDA-MB-231, BT474, MCF-7 and Hs 578Bst were obtained from the cell bank of the Chinese Academy of Sciences. SK-Br3 and MCF-10A were from ATCC. BT474, SK-Br3 and MCF-7 cells were cultured in a DMEM/F12 (1∶1 mixture; Sigma) supplemented with 10% fetal bovine serum (FBS, Hyclone), 100 U/ml penicillin and 100 mg/ml streptomycin. MDA-MB-231 cells were grown in Leibovitz-15 (L-15) medium (Invitrogen) supplemented with 10% FBS. Hs578Bst cells were cultured in DMEM/F12 supplemented with 10% FBS and EGF (30 ng/ml; Pepro Tech). Human MCF-10A non-tumorigenic mammary epithelial cells were maintained in DMEM/F-12 supplemented with 10% FBS, insulin (10 µg/ml, Invitrogen), hydrocortisone (500 ng/ml; Sigma) and EGF (20 ng/ml). MCF-10A cells are immortalized diploid cells that possess normal epithelial characteristics [39]. Cultures were maintained at 37°C in a humidified atmosphere containing 5% CO_2_.

### Protein preparation and western blot analysis

Proteins from cells or fresh tissues were extracted in RIPA lysis buffer (50 mM Tris-HCl pH 7.4, 150 mM NaCl, 1% Triton X-100, 0.25% sodium deoxycholate, 0.1% SDS) containing a protease inhibitor cocktail (Roche). Equal amounts of protein were separated by SDS-PAGE and blotted onto PVDF Immobilon-P membranes (GE Healthcare). After blocking, the membranes were probed with an anti-kin17 mAb (1∶500) overnight at 4°C. A β-actin mAb (1∶500; Santa Cruz) was used as a control for loading. Thereafter, membranes were incubated with HRP-conjugated anti-mouse IgG (1∶5000) for 45 min at room temperature. The reactions were visualized using enhanced chemiluminescence (GE Healthcare). Images were captured with ImageQuant RT ECL (GE Healthcare), and densitometry analysis was performed using ImageQuant TL 7.0 Image Analysis Software (GE Healthcare). Only samples run in the same gel were compared.

### Dual-labeled immunofluorescence

The frozen IDC tissue samples or MDA-MB-231 cells were incubated with a mixture of the anti-kin17 antibody (1∶100) and a Ki67 antibody (1∶100; Dako) overnight at 4°C. After incubation with a mixture of Alexa Fluor 488–conjugated goat anti-rabbit antibody (1∶400; Invitrogen) and Alexa Fluor 594–conjugated goat anti-mouse antibody (1∶400; Invitrogen), the slides were counterstained with 0.2 µg/ml 4′,6-diamidine-2′-phenylindole dihydrochloride (DAPI) and mounted in fluorescence mounting medium (Dako). Specimens were examined by a laser scanning confocal microscopy (Leica, TCS Sp5).

### Plasmid construction

The full-length *Kin17* ORF (nt 49–1230; GenBank accession number NM_ 012311) was amplified by PCR using PK3-Kin17, which was a kind gift from Dr. Miccoli (CEA, France). The primers were as follows: forward, 5′- ATACTCGAGATGGGGAAGTCGGATTTTC -3′; reverse, 5′- GCAAGCTTGGCAAGTTTAGAAATGTCTTCAT -3′. After digestion with *Xho* I and *Hind* III, the PCR product was inserted into the *Xho* I and *Hind* III sites of the pEGFP-N1 expression vector (Clontech), and the resulting construct was termed pEGFP-N1-Kin17. Restriction enzyme digestion and DNA sequencing were used to confirm the sequence and orientation of the recombinant construct.

### Cell transfection

Cells were transfected with siRNA_kin17 (sc-45958, Santa Cruz) or pEGFP-N1-kin17 using Lipofectamine 2000 Transfection Reagent (Invitrogen) according to the manufacturer's instructions. Control siRNA (sc-37007, Santa Cruz) or empty pEGFP-N1 vector were transfected as respective controls.

### Cell growth assay

The cells were seeded in a 24-well plate at a density of 1×10^4^ cells/ml/well and then cultured for 6 days. Cells were trypsinized and counted every 24 hr using a Coulter Counter (Beckman, Z1).

### 3-D morphogenesis assay

The cells (2.5×10^4^/well) were mixed in matrigel (BD) and added onto a 96-well plate containing an underlay of 50 µl solidified matrigel. 150 µl of culture media was then added to all wells. The phenotype of cells embedded in matrigel was determined after cultivation for 15 days.

### Soft agar colony formation

The cells (1×10^4^) were plated onto a 6-well plate containing 1% base agar and 0.5% top agar. Plated cells were incubated at 37°C for 21 days. The plates were then stained with crystal violet and colonies containing more than 50 cells were counted using ImageQuant TL 7.0 Image Analysis Software (GE Healthcare).

### EdU retention assay

Dissociated cells were exposed to 25 µM of 5-ethynyl-2′-deoxyuridine (EdU, RiboBio) for 2 hr at 37°C, and then the cells were fixed in 4% PFA. After permeabilization with 0.5% Triton-X, the cells were reacted with 1× Apollo reaction cocktail (RiboBio) for 30 min. Subsequently, the DNA contents of the cells were stained with Hoechst 33342 for 30 min and visualized under a laser scanning confocal microscopy (Leica, TCS Sp5).

### Flow cytometry

Ethanol-fixed cells were incubated with RNase A (100 µg/ml) and propidium iodide (PI, 50 µg/ml) for 30 minutes at 4°C. PI fluorescence was measured in a FACScalibur flow cytometer (BD). Data were collected from 10,000 single cell events, and cell cycle phase distributions were calculated using MODFIT software (Verity Software House).

### Alkaline comet assay

The cells were transfected with siRNA_kin17 or treated with adriamycin (ADM) at the indicated concentrations for 24 hr, whereupon DNA damage was detected using CometSlide (Trevigen, Gaithersburg, MD, USA) according to the manufacturer's instructions. After electrophoresis, we stained fragmented DNA with SYBR® Green I and visualized it under a fluorescence microscope (Nikon Eclipse Ti-U). Comet tails from 100 cells in each group were documented, and the tail DNA content was calculated using CASP Analysis Software (University of Wroclaw, Poland).

### Statistical analysis

Baseline characteristics of patients and tumors as well as tumor biology variables were compared using Student's t-test (continuous variables) and Chi-squared (χ^2^) tests (categorical variables). All statistical tests and corresponding *p*-values reported were for two-sided tests, and *p* values of less than 0.05 were considered statistically significant. SPSS (version 12.0; Chicago, IL) was used for all statistical analyses.

## Supporting Information

Figure S1 **Negative controls for immunohistochemical staining in BBD, DCIS and IDC tissues.** Scale bars = 30 µm.
(TIF)

Figure S2 **Kin17 expression in vascular endothelial cells was detected by immunohistochemistry.** Scale bars = 30 µm.
(TIF)

Figure S3 **Influence of kin17 knockdown on cell growth of MCF-10A cells.** (A) Western blot of kin17 expression in MCF-10A cells transfected with siRNA_NC or siRNA_kin17. (B) Growth curve of MCF-10A cells transfected with siRNA_NC or siRNA_kin17. This experiment was repeated at least three times, and the symbols represent the mean values of triplicate tests (mean ± SD), *p*>0.05.
(TIF)
