# Supplementary figures and images for: Up-Regulation of Kin17 Is Essential for Proliferation of Breast Cancer
Source: PLoS One. 2011 Sep 29;6(9):e25343. doi: 10.1371/journal.pone.0025343 (PMC3183049; doi:10.1371/journal.pone.0025343)

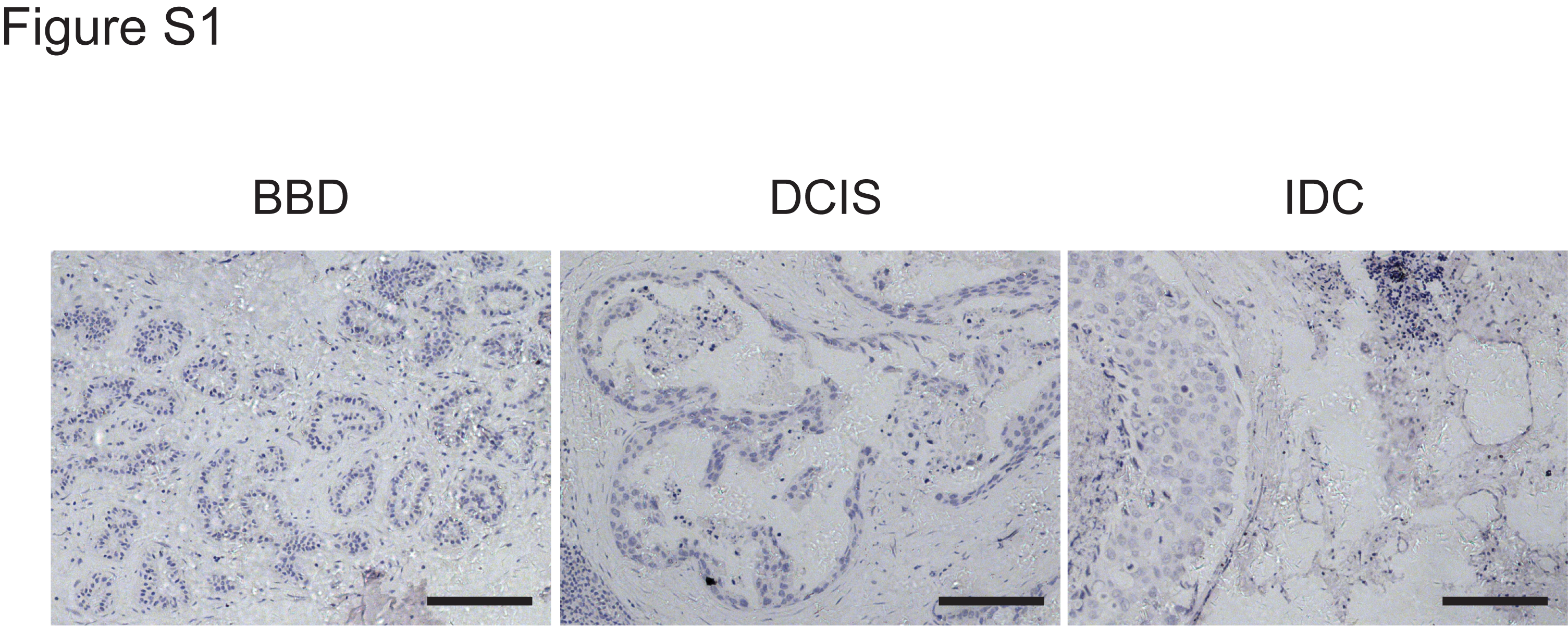

Supplement: Figure S1 — Negative controls for immunohistochemical staining in BBD, DCIS and IDC tissues. Scale bars = 30 µm. (TIF) [file pone.0025343.s001.tif]

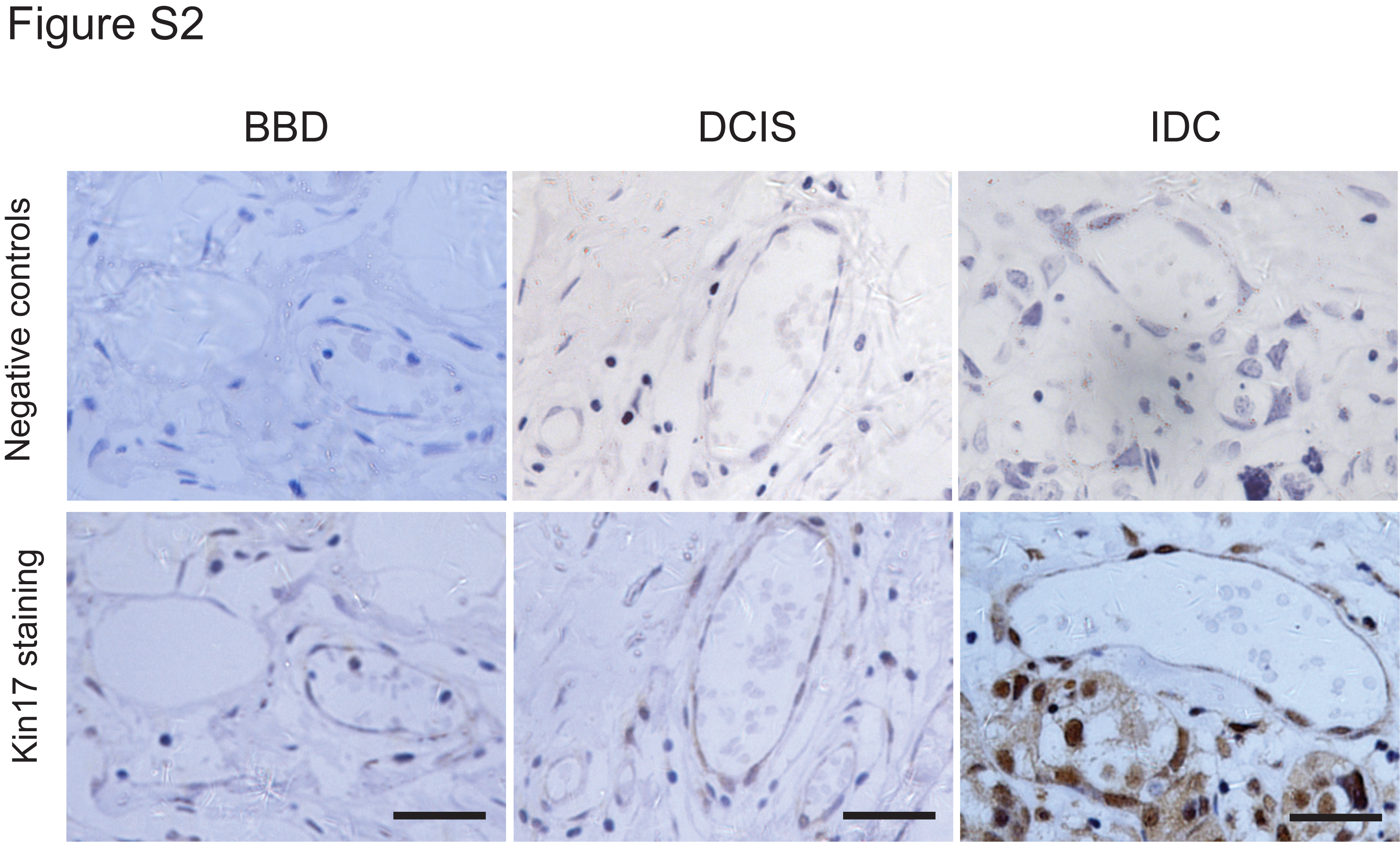

Supplement: Figure S2 — Kin17 expression in vascular endothelial cells was detected by immunohistochemistry. Scale bars = 30 µm. (TIF) [file pone.0025343.s002.tif]

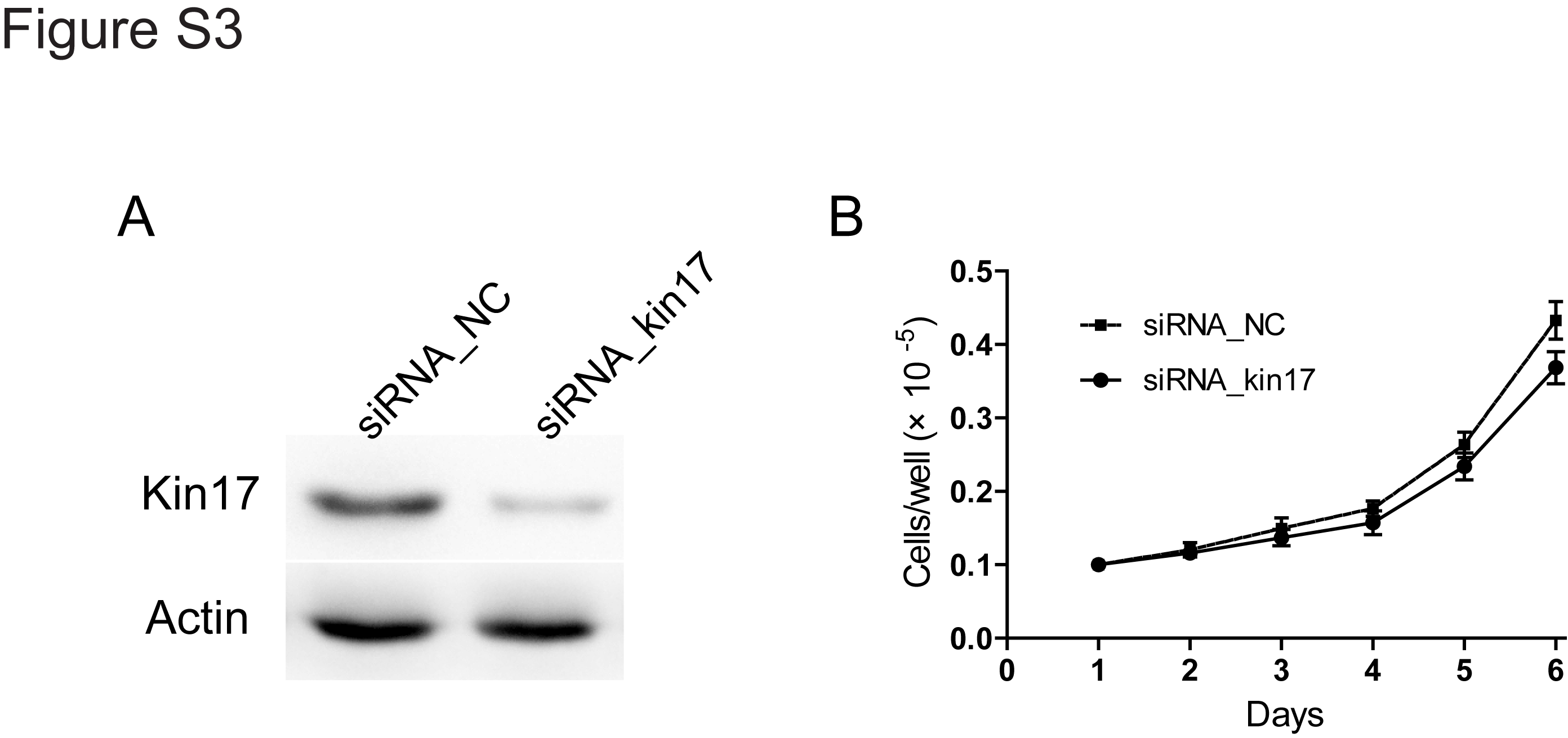

Supplement: Figure S3 — Influence of kin17 knockdown on cell growth of MCF-10A cells. (A) Western blot of kin17 expression in MCF-10A cells transfected with siRNA_NC or siRNA_kin17. (B) Growth curve of MCF-10A cells transfected with siRNA_NC or siRNA_kin17. This experiment was repeated at least three times, and the symbols represent the mean values of triplicate tests (mean ± SD), p>0.05. (TIF) [file pone.0025343.s003.tif]
